# A Murine Hypertrophic Cardiomyopathy Model: The DBA/2J Strain

**DOI:** 10.1371/journal.pone.0133132

**Published:** 2015-08-04

**Authors:** Wenyuan Zhao, Tieqiang Zhao, Yuanjian Chen, Fengbo Zhao, Qingqing Gu, Robert W. Williams, Syamal K. Bhattacharya, Lu Lu, Yao Sun

**Affiliations:** 1 Division of Cardiovascular Diseases, Department of Medicine, University of Tennessee HealthScience Center, Memphis, Tennessee, United States of America; 2 Department of Genetics, Genomics and Informatics, University of Tennessee Health Science Center, Memphis, Tennessee, United States of America; 3 Jiangsu Key Laboratory of Neuroregenertion, Nantong University, Nantong, Jiangsu, China; Albert Einstein College of Medicine, UNITED STATES

## Abstract

Familial hypertrophic cardiomyopathy (HCM) is attributed to mutations in genes that encode for the sarcomere proteins, especially *Mybpc3* and *Myh7*. Genotype-phenotype correlation studies show significant variability in HCM phenotypes among affected individuals with identical causal mutations. Morphological changes and clinical expression of HCM are the result of interactions with modifier genes. With the exceptions of angiotensin converting enzyme, these modifiers have not been identified. Although mouse models have been used to investigate the genetics of many complex diseases, natural murine models for HCM are still lacking. In this study we show that the DBA/2J (D2) strain of mouse has sequence variants in *Mybpc3* and *Myh7*, relative to widely used C57BL/6J (B6) reference strain and the key features of human HCM. Four-month-old of male D2 mice exhibit hallmarks of HCM including increased heart weight and cardiomyocyte size relative to B6 mice, as well as elevated markers for cardiac hypertrophy including β-myosin heavy chain (MHC), atrial natriuretic peptide (ANP), brain natriuretic peptide (BNP), and skeletal muscle alpha actin (α1-actin). Furthermore, cardiac interstitial fibrosis, another feature of HCM, is also evident in the D2 strain, and is accompanied by up-regulation of type I collagen and α-smooth muscle actin (SMA)-markers of fibrosis. Of great interest, blood pressure and cardiac function are within the normal range in the D2 strain, demonstrating that cardiac hypertrophy and fibrosis are not secondary to hypertension, myocardial infarction, or heart failure. Because D2 and B6 strains have been used to generate a large family of recombinant inbred strains, the BXD cohort, the D2 model can be effectively exploited for in-depth genetic analysis of HCM susceptibility and modifier screens.

## Introduction

Hypertrophic cardiomyopathy is a common heart disease that affects about one out of 500 humans. It occurs in a non-dilated ventricle in the absence of other cardiac pathologies capable of producing the observed magnitude of increased left ventricular wall thickness, such as hypertension, myocardial infarction, heart failure, etc. [1–3]. HCM is most commonly associated with a mutation of sarcomeric genes that results in a mutated protein in the sarcomere, the primary component of cardiomyocytes [4]. These are predominantly single-point missense mutations in the genes for myosin-binding protein C (*Mybpc3*), β-MHC *(myh7)*, cardiac troponin T (*Tnnt2*), etc. [5]. In individuals without a family history of HCM, the most common cause of the disease is a de novo mutation of the gene that produces β-MHC.

Clinical phenotypes of HCM in humans include left ventricular hypertrophy and diminished left ventricular cavity size [6, 7]. Microscopic features include cardiomyocyte hypertrophy and disorganization, myocardial fibrosis and abnormal intramural coronary arteries with thickened walls and perivascular fibrosis [8]. Molecular phenotypes primarily comprise up-regulation of hypertrophic and fibrotic markers, including β-MHC, α1-actin, ANP, type I collagen, α-SMA, etc. [9].

The intriguing feature of HCM is its broad spectrum of clinical manifestations that range from benign, asymptomatic hypertrophy, heart failure, and sudden death [7, 10]. Even among patients with mutations in the same gene, there is considerable variation in symptoms and severity. Marian and colleagues have emphasized that a second tier of genetic modifier loci contribute to this clinical diversity [11]. Therefore, although a HCM-causing mutation has been identified, the genetic modifiers are considered to account for the clinical diversity. HCM phenotype modifiers, however, remain largely unidentified and are likely to depend on the interaction of multiple genes [11].

Identification of modifier genes that cause variation in HCM risk in humans has proven difficult. Although human genome-wide association studies (GWAS) are now feasible, the power of these studies is modest and difficult to tightly control the environmental variables. So far only one GWAS study of HCM has been published [12]. Animal study can overcome certain limitations of human research and create opportunities to investigate both disease mechanisms and potential therapies. However, suitable model for inheritable HCM is currently unavailable.

In this study we have characterized sequence variants in the genes for myosin-binding protein C (*Mybpc3*) and β-MHC *(myh7)* in the D2 strain of mouse compared to the B6 reference strain. The D2 strain reveals many of the hallmarks of HCM without overt signs of other cardiac disease such as hypertension, cardiac damage or heart failure. In contrast, the B6 strain has no distinguishable cardiovascular pathology. This new model of HCM holds great promise. D2 and B6 are parents of a large set of recombinant inbred strains—the BXD cohort [13]—that can now be exploited to test genetic mechanisms of HCM susceptibility, progression, and intervention.

## Materials and Methods

### Animals

In the current study, four-month-old-male B6 and D2 strains (n = 15/strain) were used, which were born and raised in our vivarium at the University of Tennessee Health Science Center. Mice were housed 3–4 per cage in a temperature-controlled room (22°C), maintained at a 12h light/dark cycle and fed a standard chow (Harlan-Teklad, #7912). The animal protocol was approved by the Animal Care and Use Committee of University of Tennessee Health Science Center.

### Cardiomyocyte Hypertrophy

Cardiac hypertrophy was evaluated by determining the heart weight, cardiomyocyte size and the expression of cardiac hypertrophic markers.

#### Cardiomyocyte Size

Cryostat sections of the heart (6μm) were collected from the apex, mid cavity, and base and stained with hematoxylin and eosin. Morphometric analysis of cardiomyocyte size was performed on cardiac sections using an image quantitative digital analysis system (NIH Image 1.6). Single cardiomyocyte size was measured with images captured from these sections. Cardiomyocyte cross‐sectional diameter of 100 to 200 cardiomyocytes was measured from each section [14].

#### Cardiac Hypertrophy Markers

Cardiac β-MHC, ANP, BNP and α1-actin are well-recognized markers of cardiac hypertrophy. The gene expression of these hypertrophic markers was investigated by RNA-seq. In brief, total RNA from hearts of B6 and D2 mice was extracted using RNeasy kit. The Sense mRNA-seq Library Prep Kit for Ion Torrent was used for Poly A purification and library generation. The library was amplified to add the barcode adapter sequences and to generate sufficient material for sequencing. Barcoded samples were then sequenced on an Ion Proton instrument, using P1 chips and the V2 sequencing kit. Partek Flow and other software (Lifescope, Deseq R codes, python scripts, etc.) were used for alignments, quantification and statistical analysis [15].

Cardiac β-MHC and α1-actin mRNA levels detected by RNA-seq in B6 and D2 strains were validated by quantitative PCR (qPCR) as we previously reported [16].

Cardiac β-MHC protein levels in both B6 and D2 mice were further confirmed by western blot as reported by us previously [17].

### Cardiac Interstitial Fibrosis

Cardiac fibrosis was examined by expression of fibrotic markers, quantifying collagen volume fraction and expression of fibrotic markers.

#### Cardiac Fibrosis Markers

Type I collagen is the major collagen isoform present in the fibrous tissue. Myofibroblasts play a primary role in cardiac fibrosis in the diseased/damaged heart. The hallmark of myofibroblasts is their expression of α-SMA [18, 19]. Thus, type I collagen and α-SMA serve as markers of cardiac fibrosis. Type I collagen and α-SMA gene expression was detected by RNA-seq. Cardiac α-SMA protein level was further assessed by western blot as we previously reported [17].

#### Cardiac Collagen Volume Fraction

Cryostat cardiac sections (6μm) were prepared to evaluate the appearance of interstitial fibrosis by collagen-specific picrosirius red staining and examined by light microscopy. Collagen volume fraction was determined using a computer image analyzing system and calculated as the sum of connective tissue area, divided by the sum of connective tissue area and non-connective tissue area in all fields of the heart section (4 sections/heart) as we previously reported [20].

#### Cardiac Myofibroblasts

Appearance of myofibroblasts in the heart was detected using immunohistochemical α- SMA staining. In brief, cardiac sections (6μm) were incubated with working solution of mouse on mouse Ig blocking reagent (Vector laboratory, Burlingame, CA) for 1 hour, followed by the primary antibody against α-SMA (Sigma, St Louis, MO) for 1 hour at room temperature. The sections were then incubated with the IgG-peroxidase-conjugated secondary antibody (Sigma, St Louis, MO) for 1 hour at room temperature, and incubated with 0.5 mg/ml diaminobenzidine tetrahydrochloride 2-hydrate + 0.05% H_2_O_2_ for 5 min. Negative control sections were incubated with the secondary antibody alone. Myofibroblasts in the interstitials space and smooth muscle cells of blood vessels in the cardiac sections should be positively labelled.

### Blood Pressure

Blood pressure was measured using tail cuff. Mice were maintained in still and unperturbed position throughout the measurement period. Mice were conditioned to the restraint and the warming chamber for 10–20 min/day for 3 days before measurements. The computerized blood pressure monitor was set for desired sensitivity, number of cycles, the maximum tail-cuff inflation pressure, the rate of deflation and the interval between cycles [21].

### Ventricular Function

Ventricular function was examined by echocardiography. Mice were lightly anesthetized with isoflurane at a constant volume and anesthetic time. Transthoracic echocardiography was performed in the left lateral decubitus position using the echocardiographic system (Sonos 4500 with a 7- to 11-MHz transducer). The parasternal short-axis view under two-dimensional M-mode at the level of the papillary muscle was recorded. Ejection fraction (EF), left ventricular diastolic diameter (LVDd), left ventricle systolic diameter (LVDs), left ventricular diastolic posterior wall thickness (LVPWd), left ventricular systolic posterior wall thickness (LVPWs), left ventricular diastolic anterior wall thickness (LVAWd), and left ventricular systolic anterior wall thickness (LVAWs) were calculated in accordance with the American Society of Echocardiography guidelines as we previously reported [22].

### Cardiac Morphology

Cardiac morphology in B6 and D2 strains was examined by hematoxylin and eosin staining and viewed by the light microscope.

### Statistical Analysis

Statistical analysis of cardiomyocyte size, RNA-seq, qPCR, western blot, collagen volume fraction, blood pressure and ventricular function data between B6 and D2 strains was performed using student *t* test. Values are expressed as mean±SEM, and *p*<0.05 considered statistically significant.

## Results

### *Myh7 and Mybpc3* Sequence Variants in the D2 Strain

Patients with a high index of clinical suspicion for HCM have a mutation identified in at least 1 of 9 sarcomeric genes. Approximately 40% of these mutations occur in the the cardiac myosin binding protein C gene (*Mybpc3*), while about 40% involve β-MHC (*Myh7*). Based on the sequence of B6 and D2 strains, we found that D2 mice had sequence variants in *Mybpc3* and *Myh7*, compared to B6 mice. The D2 strain had nonsynomous SNPs and start-loss variants in *Mybpc3* and a start-gain variant in *Myh7*,in comparison with the B6 strain (Table 1).

**Table 1:** Polymorphism on *Myh7* and *Mybpc3* Genes between B6 and D2 Strains.

Gene	Chr	Mb	Alleles	Domain	Function	B6	D2
*Myh7*	14	55.612192	A/G	5' UTR	Start Gained	A	G
*Mybpc3*	2	90.958328	G/A	Coding	Start Lost	G	A
*Mybpc3*	2	90.959360	C/T	Coding	Nonsynonymous	C	T
*Mybpc3*	2	90.959508	A/G	Coding	Nonsynonymous	A	G
*Mybpc3*	2	90.960171	G/A	Coding	Nonsynonymous	G	A
*Mybpc3*	2	90.969172	A/G	Coding	Nonsynonymous	A	G

### Cardiac Hypertrophy

#### Heart Weight, Cardiomyocyte Size and Wall Thickness

**Fig 1:**
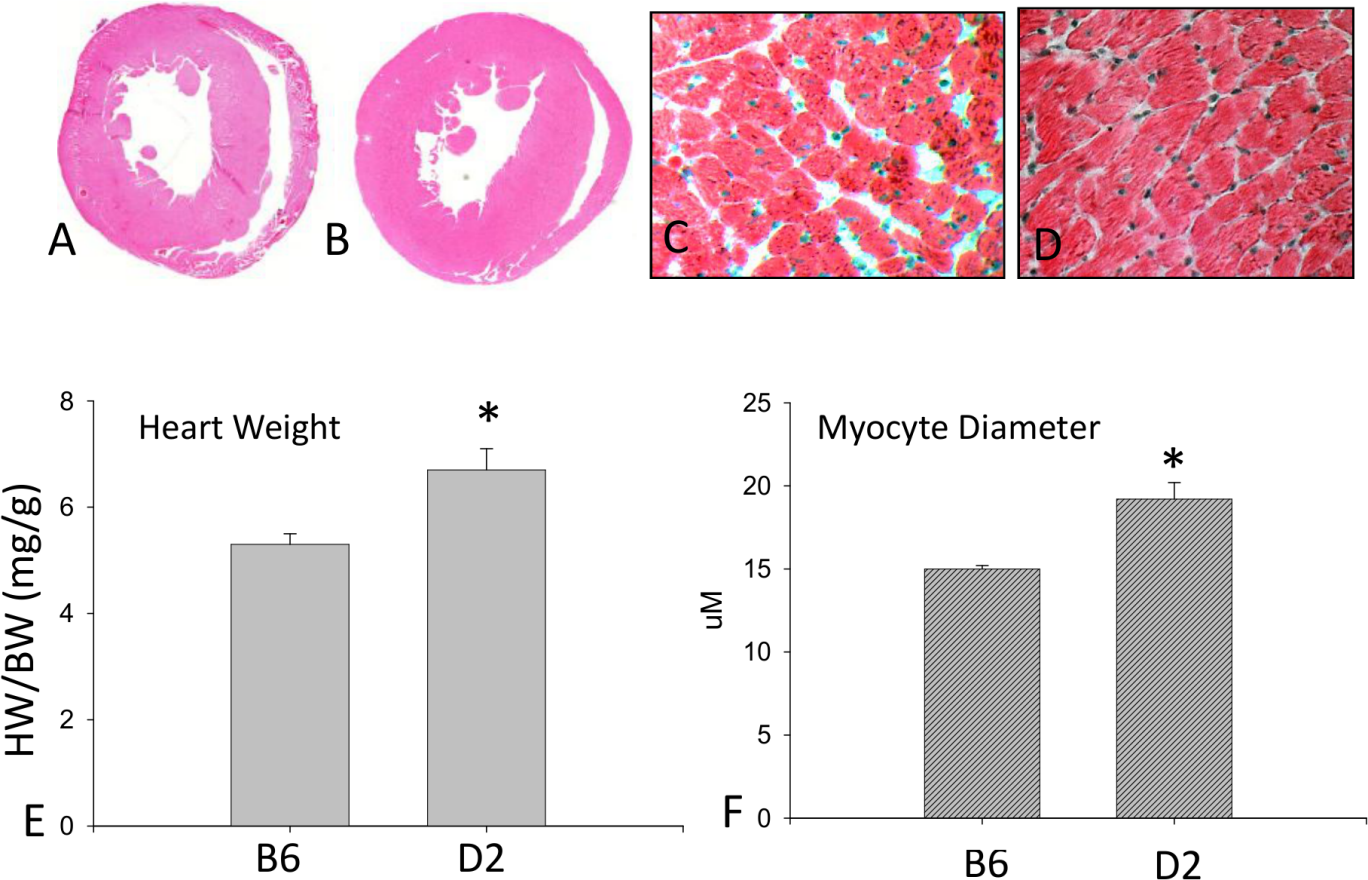
Heart weight and cardiomyocyte size. Left ventricle thickness (panel B) and cardiomyocyte size (panel D) were markedly increased in D2 mice compared to B6 mice (panels A and C, respectively). Heart weight (panel E) and cardiomyocyte diameter (panel F) were significantly enhanced in the D2 strain compared to the B6 strain. Panels C and D: x200

Compared to the B6 strain, left ventricular wall thickness and heart weight were consistently greater in the D2 strain (Fig 1A, 1B and 1E). Furthermore, cardiomyocyte size and diameter in the D2 mice was markedly increased relative to B6 mice (Fig 1C, 1D and 1F).

**Fig 2:**
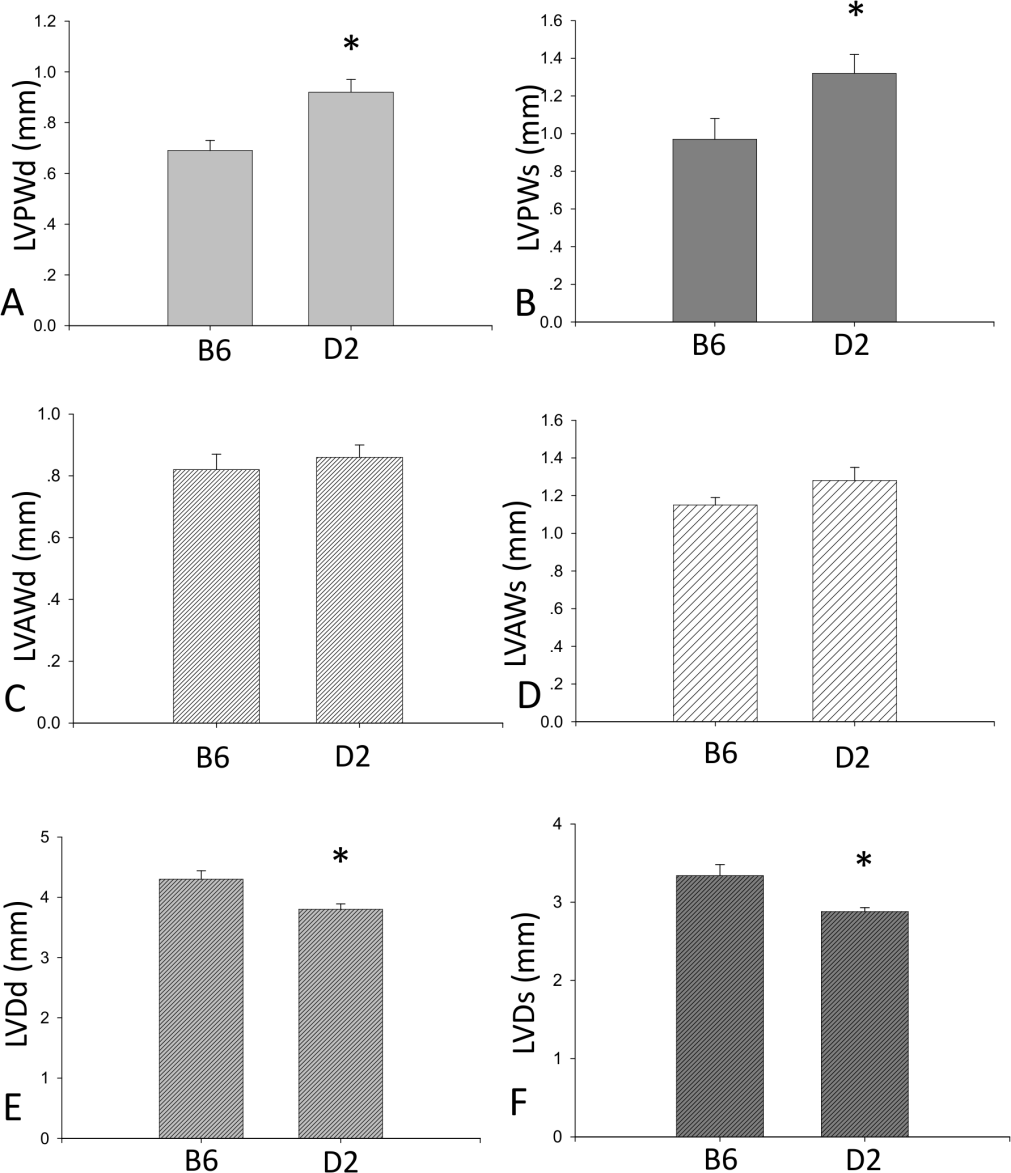
Echocardiography determination of cardiac dimensions. Compared to B6 mice, LVPWd (panel A), LVPWs (panel B), LVDd (panel E) and LVDVs (panel F) were significantly greater in D2 mice. There was no difference in LVAWd (panel C) and LVAWs (panel D) between B6 and D2 mice.

As detected by echocardiography, LVPWd and LVPWs were significantly increased, while LVDd and LVDs were significantly reduced in D2 mice compared to B6 mice. However, there was no appreciable difference in LVAWd and LVAWs between B6 and D2 strains (Fig 2).

#### Expression of Cardiac Hypertrophic Markers

**Fig 3:**
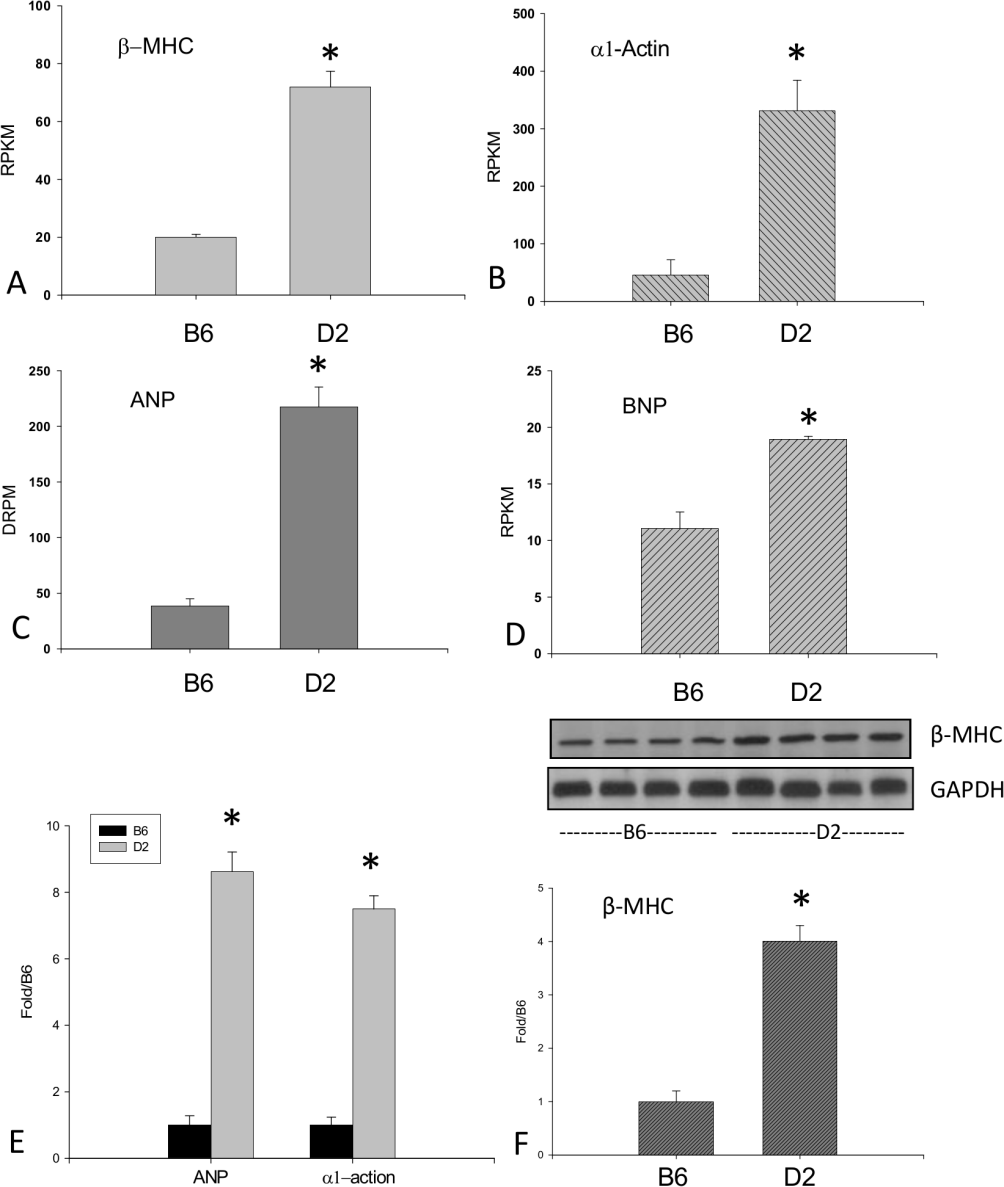
Cardiac hypertrophy in the D2 strain. As detected by RNA-seq, β-MHC, α1-actin, ANP and BNP mRNA levels were significantly elevated in D2 mice compared to B6 mice (panels A-D). RNA-seq data on ANP and α1-actin were validated by qPCR (panel E). Western blot analysis further revealed that β-MHC protein levels in the D2 strain were significantly greater than the B6 strain (panel F).

As detected by RNA-seq, we observed significantly increased cardiac hypertrophic markers, including β-MHC, α1-actin, ANP and BNP mRNA levels in the D2 strain compared to B6 mice (Fig 3A–3D). RNA-seq data on ANP and α1-actin mRNA were further validated by qPCR (Fig 3E).

Cardiac β-MHC protein level was further detected by western blot. Cardiac β-MHC levels in D2 mice were significantly increased in comparison with B6 mice (Fig 3F).

### Cardiac Interstitial Fibrosis

#### Expression of Markers Associated with Fibrosis

**Fig 4:**
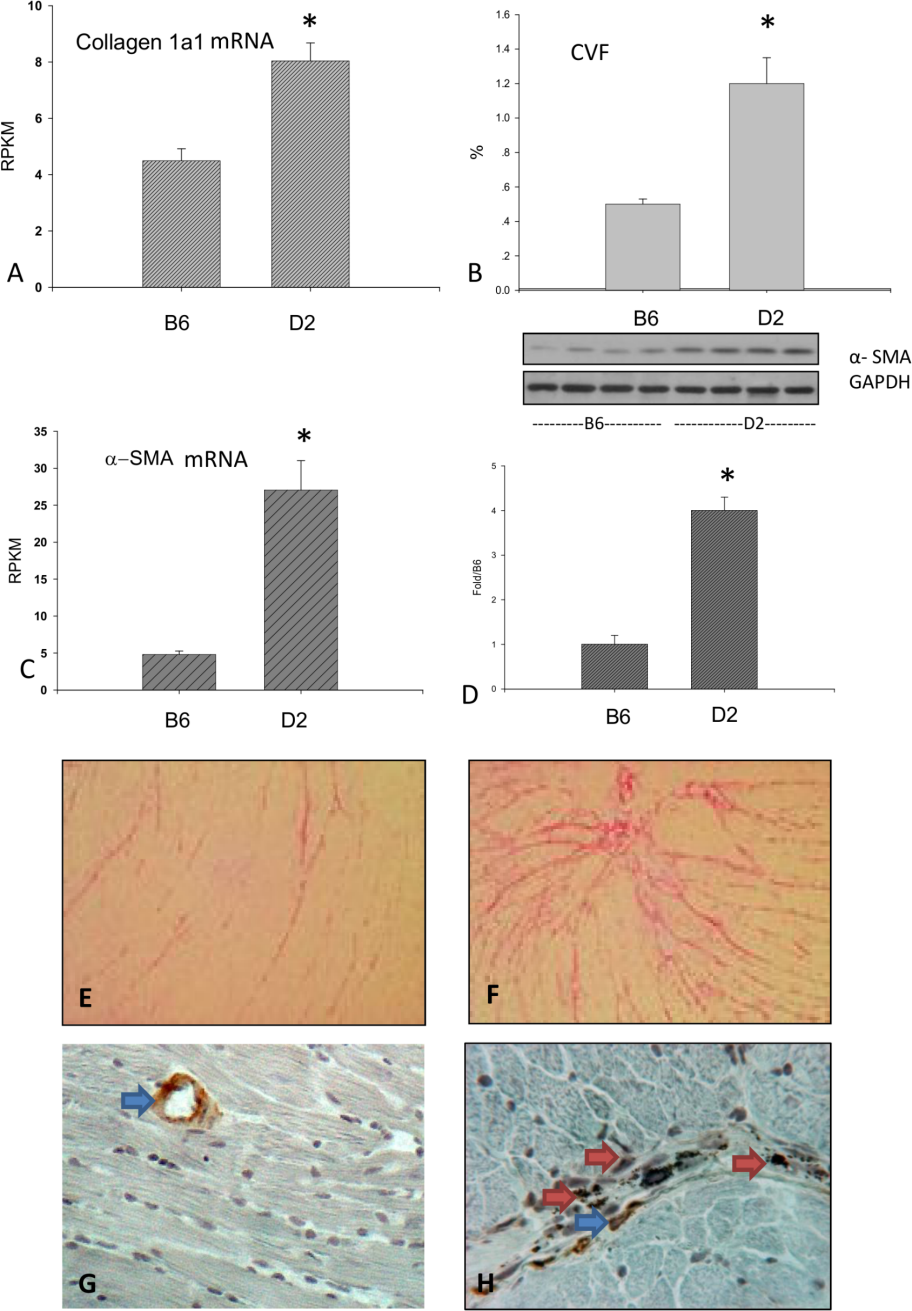
Cardiac fibrosis in the D2 strain. Cardiac type I collagen and α-SMA mRNA (panels A and C), collagen volume fraction (panel B), and α-SMA level (panel D) in the D2 strain were significantly greater compared to the B6 strain. A small amount collagen was present in the interstitial space in B6 mice (panel E). Cardiac interstitial fibrosis was, however, developed in D2 mice (panel F). α-SMA^+^ myofibroblasts were not present in cardiac interstitial space of B6 mice (panel G, blue arrow: α-SMA^+^ vascular smooth muscle cells), while myofibroblasts were accumulated in the interstitial space of the left ventricle in D2 mice (panel F, red arrows). Panels E-H: x200

As illustrated in Fig 4A and 4B, the gene expression of type I collagen and α-SMA was significantly elevated in D2 mice compared to B6 mice. Furthermore, cardiac α-SMA protein levels in D2 mice were significantly higher than in B6 mice (Fig 4F).

#### Cardiac Collagen Volume

As detected by collagen specific picrosirius red staining, a small amount of collagen was present in the normal myocardium of B6 mice (Fig 4C). Cardiac interstitial collagen deposition was greater in D2 mice relative to B6 mice (Fig 4D). The quantitative cardiac collagen volume fraction data for B6 and D2 mice are shown in Fig 4F.

#### Cardiac Myofibroblasts

Myofibroblasts are activated fibroblasts and appear in the repairing tissue [20]. By immunohistochemical α-SMA labeling, myofibroblasts were not observed in the heart of B6 mice (Fig 4G). Numerous myofibroblasts, however, were observed in the cardiac interstitial space of the D2 strain (Fig 4H), which was co-localized with accumulated collagen. Smooth muscle cells of blood vessels also contain α-SMA and were positively labeled in the cardiac sections of B6 and D2 mice (Fig 4G and 4H).

### Blood Pressure

**Fig 5:**
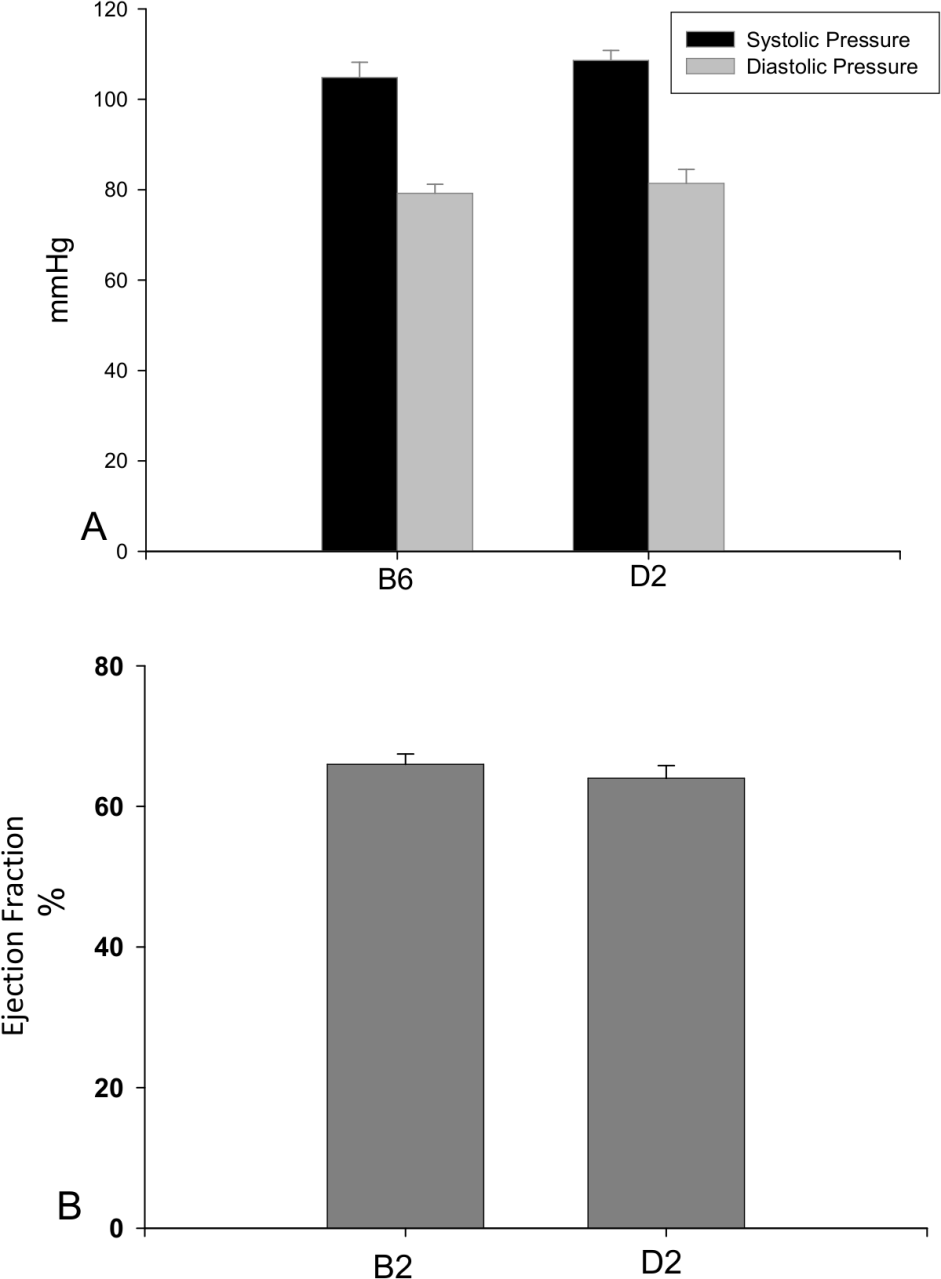
Blood pressure and ventricular function. Blood pressure (panel A) and ventricular function (panel B) were in the normal range in both B6 and D2 mice.

The major clinical feature of HCM is the increased left ventricle wall thickness without other systemic and cardiac diseases, particularly hypertension. To determine whether cardiac hypertrophy and interstitial fibrosis in D2 mice are related to hypertension, we monitored blood pressure in B6 and D2 strains and found that systolic and diastolic blood pressure was normal in both strains (Fig 5A).

### Ventricular Function

We further examined whether cardiac hypertrophy and fibrosis in the D2 strain were associated with heart failure. Our echocardiographic study revealed that ejection fraction was normal in D2 mice, which was not different from that in B6 mice (Fig 5B).

### Cardiac Morphology

Cardiac damage, particularly induced by myocardial infarction, is another cause of cardiac hypertrophy. However, our cardiac morphological study demonstrated that D2 mice did not have any sign of myocardial infarction (Fig 1D).

## Discussion

B6 and D2 strains are two of the most commonly used inbred mice in medical research. The B6 strain has been sequenced more than ten years ago and is the reference sequence for mouse genome [23]. Recently, the D2 strain has been fully sequenced [24, 25]. Based on our analysis of the sequence data, these two strains differ at approximately 4.8 million single nucleotide polymorphisms. The characteristics of the D2 strain are often contrasted with those of the B6 strain. Studies have shown that due to specific gene mutations, the D2 strain has a greater susceptibility to age related hearing loss and glaucoma, audiogenic seizures, as well as calcified lesions of the testes, tongue, skeletal muscle, etc. [26–28].

B6 mice have the normal cardiac structure and ventricular function. Based on the gene sequence of B6 and D2 strains, the D2 strain contain sequence variants in *Mybpc3* and *Myh7* genes, the major causal genes involving in HCM. HCM is induced by a mutation in at least 1 of 9 sarcomeric genes in human. Among these, approximately 80% of HCM is induced by *Mybpc3* and *Myh7* mutations [29]. Deletion of *Mybpc3* in B6 mice has been reported to lead to HCM phenotypes [30]. Thus, D2 mice have the genetic basis of HCM.

Next, we examined whether *Mybpc3* and *Myh7* sequence variants in the D2 strain are accompanied by HCM phenotypes. Cardiac hypertrophy is the major clinical HCM phenotype induced by increased cardiomyocyte size, resulting in the thickening of the heart muscle. Our study reveals that the heart weight and left ventricular wall thickness in the D2 strain are significantly greater than the B6 strain. Furthermore, cardiomyocyte size and a series of cardiac hypertrophic markers in the D2 strain are significantly increased relative to the B6 strain, implicating that cardiac hypertrophy is spontaneously developed in the D2 strain. In addition, cardiac hypertrophy in HCM is usually asymmetric. Our echocardiographic data further demonstrated that left ventricular hypertrophy is uneven in D2 mice, which corresponds to the nature of cardiac hypertrophy in HCM.

Myocardial fibrosis is another important feature of HCM and a proposed substrate for arrhythmias that often appears in HCM [31]. Interstitial fibrosis, which surrounds individual cardiomyocytes, and focal fibrosis, the microscopic scars that replace dead myocytes, continue to accumulate in HCM. Myofibroblasts are phenotypically transformed fibroblasts, which play a central role in fibrous tissue deposition in the heart with HCM. Expression of α-SMA is a feature of myofibroblasts. Our study reveals significantly increased cardiac type I collagen (major component of fibrous tissue) and α-SMA expression in D2 mice compared to B6 mice. The quantitative data further show the increased cardiac collagen volume in D2 mice. The increased cardiac fibrosis is coincident with the appearance of myofibroblasts. These observations suggest that cardiac interstitial fibrosis is spontaneously developed in D2 mice.

In addition to HCM, cardiac hypertrophy and interstitial fibrosis also appear in other cardiac diseases, particularly increased afterload (e.g. systemic hypertension), myocardial infarction, heart failure etc. In patients with HCM, cardiac hypertrophy and fibrosis occur without any obvious cause. The current study has shown that blood pressure and ventricular function remain normal in D2 mice, implicating that cardiac pathology in the D2 strain is not associated to hypertension and heart failure. Furthermore, myocardial infarction is not observed in D2 mice, indicating that cardiac hypertrophy and fibrosis in the D2 strain are not secondary to cardiac injury. HCM causes progressive ventricular dysfunction and exhibit increased mortality as they age. In the future study, we will further investigate clinical and laboratory phenotypes of HCM in the D2 strain at the different age.

The severity and penetrance of HCM greatly varies among patients with HCM gene mutations. Some have shortness of breath, chest pain, heart failure, and even sudden death; others are mildly symptomatic; while in other, these mutations never develop any disease. Factors causing variability in HCM disease penetrance and expression are poorly understood at this time. Discovering of the genetic basis of differential vulnerability to HCM is critical in predicting and developing personalized care for patients and will provide the genetic determinants of cardiac phenotypes in HCM, enabling comprehensive genetic screening with an aim to eliminate sudden death. The genetic modifiers are considered to account for the clinical diversity. Angiotensin converting enzyme gene has been recognized to serve as a modifier gene [32, 33]. Other HCM phenotype modifiers, however, remain largely unidentified and are likely to depend on the interaction of multiple genes [34].

Elucidation of modifier genes that cause variation in HCM risk in humans has proven difficult. Animal study can overcome the limitations of human research and create opportunities to investigate both disease mechanisms and potential therapies. Large murine genetic reference populations (GRPs) are one of the best-established mammalian models to study complex trait and genes that cause or affect diseases in humans [35, 36]. The BXD family, currently the largest and best characterized mouse GRP, is composed of over 100 lines that descend from crosses between B6 and D2 parents. This resource has significant power to dissect the molecular architecture of complex traits and gene function. BXD strains and their parent strains, B6 and D2 mice, therefore, are well-positioned to investigate HCM genetic background (modifier genes).

Building upon the genotype and cardiac phenotypes of B6, D2, and BXD strains, we will identify genes that modulate variability of HCM phenotypes using system genetic strategy in our future study.

In summary, this is the first study showing that the D2 strain has both HCM genotype and phenotypes, indicating that D2 mice serve as a suitable model for HCM investigation.
